# Effect of GDM Pairing on PEMFC Performance in Flow-Through and Dead-Ended Anode Mode

**DOI:** 10.3390/molecules25061469

**Published:** 2020-03-24

**Authors:** Yannick Garsany, Cornelius H. Bancroft, Robert W. Atkinson III, Keith Bethune, Benjamin D. Gould, Karen E. Swider-Lyons

**Affiliations:** 1 EXCET, Inc., Springfield, VA 22151, USA; 2 US Naval Research Laboratory, Washington, DC 20375, USA; 3 Hawaii Natural Energy Institute, University of Hawaii, Honolulu, HI 96822, USA

**Keywords:** PEMFCs, asymmetric & symmetric GDM, Freudenberg, SGL 29BC, dead-ended anode (DEA) mode

## Abstract

Asymmetric gas diffusion media (GDM) pairing, which feature distinct GDM at the anode and cathode of the proton electrolyte membrane fuel cell (PEMFC), enhance water management compared to symmetric pairing of GDM (anode and cathode GDM are identical). An asymmetric pairing of Freudenberg GDM (H24C3 at anode and H23C2 at cathode) reduces ohmic resistances by up to 40% and oxygen transport resistances by 14% en route to 25% higher current density in dry gas flows. The asymmetric GDM pairing effectively hydrates the membrane electrode assembly (MEA) while minimizing liquid water saturation in the cathode compared to a commonly used symmetric GDM pairing of SGL 29BC at the anode and cathode. Superior water management observed with asymmetric GDM in flow-through mode is also realized in dead-ended anode (DEA) mode. Compared to the symmetric GDM pairing, the asymmetric GDM pairing with Freudenberg GDM increases cell voltage at all current densities, extends and stabilizes steady-state voltage behavior, slows voltage decay, and vastly reduces the frequency of anode purge events. These results support that the asymmetric Freudenberg GDM combination renders the PEMFC less prone to anode water saturation and performance loss from the anticipated increase in water back-diffusion during DEA mode operation.

## 1. Introduction

Proton exchange membrane fuel cells (PEMFCs) are clean electrochemical power sources for use in a broad array of applications [1]. System costs are still the major challenge to widespread commercialization of PEMFCs. One approach to reduce cost and system complexity is to operate the PEMFC in dead-ended anode (DEA) mode. Operating a PEMFC in DEA mode simplifies the balance of plant requirements by removing the hydrogen ejector/blower, gas humidifier, mass flow meter, and redundant piping [2,3,4].

During the DEA operation of a PEMFC, hydrogen is supplied to the inlet of the DEA PEMFC system and a normally closed solenoid valve blocks the outlet. Using this simple set-up reduces the system cost and increases the PEMFCs hydrogen utilization [5,6]. There are two major failure modes for the voltage decay in PEMFCs operated in DEA mode: dilution of the anode fuel concentration via N_2_ crossover and excessive accumulation of back diffused liquid water from the cathode. Both of these failure modes can be exacerbated by improper materials selection inside the PEMFC. Liquid water generated at the cathode can back diffuse through the membrane and accumulate inside the anode gas diffusion media (GDM) and flow channels [7,8,9,10,11,12], blocking the gas transport pathway. When air is supplied as the oxidant, N_2_ can diffuse through the membrane due to a pressure concentration gradient [7,12,13,14,15], resulting in local fuel (i.e., H_2_) starvation and performance loss. 

A typical PEMFC is composed of bipolar plates (BPPs) and a membrane electrode assembly (MEA), which is in a five layer structure comprising a proton exchange membrane (PEM) at the center, two coated catalyst layers (CLs) and two GDM [16]. The GDM are essential components of the PEMFCs, and play multiple functions during the PEMFC operation such as transportation of reactant gases to the CLs, removing produced water and heat from the MEA to the BPPs, and conducting electrons from the CLs to the BPPs [17,18,19]. GDM typically consist of porous carbon matrices (cloths, papers, or nonwovens) and are comprised by two regions. The fibrous gas diffusion layer (GDL) substrate has larger pores or voids and serves as a relatively robust substrate that gives the MEA its mechanical integrity. The GDL is comprised of carbon for electrical conductivity and is commonly treated with fluoropolymers (i.e., PTFE) for hydrophobicity. A carbon particle-based, hydrophobic, microporous layer (MPL) is coated on the GDL to improve thermal and electrical contact with the CLs, and maintain humidification of the adjacent CLs and the PEM while avoiding flooding of its porous backing (the GDL substrate), which would compromise the reactant gas supply to the CLs [20,21,22].

To date, the vast majority of the PEMFC open literature focuses on testing the PEMFCs using a symmetric GDM pairing, i.e., the same GDM is used on the anode and cathode side of the MEA. However, when selecting GDM for PEMFCs, it is critical to consider the anode and cathode GDM properties in tandem as each electrode plays a role in global cell water management. Careful pairing of the two GDM in concert can have a tremendous impact on the PEMFC performance. Recently, Schweiss reported that PEMFCs tested using an asymmetric GDM pairing, with a distinct anode GDM that improves water retention and a porous cathode GDM that promotes high oxidant diffusivity, are less sensitive to relative humidity and increases PEMFC current density compared to all tested symmetric GDM configurations (same GDM used on the anode and cathode) [23]. We have previously shown in open-cathode fuel cells that an asymmetric GDM pairing featuring higher porosity in the anode GDM than the cathode significantly improves hydration and power production [24,25].

In this work, we compare an asymmetric GDM pairing utilizing Freudenberg GDM (H24C3 at anode, H23C2 at cathode) to a symmetric GDM pairing frequently used in the open literature containing SGL 29BC at both the anode and cathode, to highlight the impact of the GDM water management on fuel cell operation in flow-through mode and DEA mode in a range of cathode inlet relative humidity.

## 2. Materials and Methods

### 2.1. PEMFC Performance in Regular Flow-Through Mode

Two distinct anode and cathode GDM pairings were tested in this study: symmetric and asymmetric. The symmetric GDM pairing was comprised of SGL 29BC carbon paper (SIGRACET, SGL Carbon Inc.) at both the anode and cathode, which consisted of 5% PTFE loading in the GDL and 23% PTFE loading in the MPL. The asymmetric GDM pairing investigated in this study utilized H23C2 (Freudenberg FCCT SE.) carbon felt on the cathode side, which was not PTFE-treated in the GDL and had 40% PTFE loading in the MPL. The anode side contained H24C3 (Freudenberg FCCT SE & Co.), which was PTFE-treated in the GDL and MPL.

Fuel cells were tested in a 25 cm^2^ active area single cell hardware (Fuel Cell Technologies) with individually designed graphite flow fields (Poco Graphite), which comprised mirror symmetric flow patterns for the anode and the cathode. The flow fields consisted of 33 parallel channels with a channel and land width of 1 mm and a channel depth of 0.9 mm, which were arranged in triple parallel serpentine.

Membrane electrode assemblies (MEAs) were prepared by combining the Freudenberg GDM with a Primea^®^ MESGA catalyst coated membrane (CCM, W. L. Gore & Associates A510.4/M710.18/C510.4, containing sub-gaskets) with Pt loading of 0.40 mg_Pt_ cm^−2^ on the anode side and 0.40 mg_Pt_ cm^−2^ on the cathode side, respectively. The square active electrode area was 25 cm^2^, while the GDM were 27.04 cm^−2^. When perfectly aligned, the GDM extended ~0.2 cm beyond the electrode active area on all sides.

All Freudenberg GDM were compressed by 28% of the initial uncompressed thickness, which we found to be optimal in our prior work [26]. The SGL 29BC GDM were compressed to 14% of the initial uncompressed GDM thickness, the optimal value for these materials in these operating conditions [26,27]. In order to compress the MEA to achieve the desired GDM compression, the thicknesses of the CCM and GDM were measured with a digimatic micrometer (Mitutoyo, Model MDC-1” PX) at 9 evenly spaced locations over each component area. These measurements were averaged to calculate the component thickness and used to determine the required gasket thickness. PTFE Skived Tape (Enflo) were placed on the anode and cathode side of the membrane to enable the desired GDM compression [16,26,27]. The final fuel cell assembly was sealed with 8 bolts torqued to 10 Nm per bolt in a star pattern. 

Once assembled, the performance of single cells was tested using Scribner 850e Fuel Cell Test Systems from Scribner Associates, Inc. All experiments were conducted at 65 °C and ambient pressure (1 atm), unless otherwise noted. Humidifiers were filled with 18 MΩ cm water from a Barnstead Nanopure System. The inlet dew point for both gases was set at 50.3 °C and 37 °C, corresponding to 50% and 25% inlet relative humidity (RH), respectively. Ultrapure gases, H_2_ (HY UHP300) or Air (UZ 300), purchased from Air-liquide, were supplied to the anode and cathode, respectively, under stoichiometric flow conditions 2|2 for H_2_|air, unless otherwise noted. All experiments started by pre-conditioning the PEMFC with the following sequence: the cell voltage was first held at 0.60 V in H_2_|air for 2 h, followed by 20 cycles that alternated between 0.70 V and 0.40 V with each voltage held for 10 min. The cell and gas temperature used in the “break-in” procedure were used to collect the current-voltage (I–V) polarization curves. I–V polarization curves were recorded at increments of 25 mV from open circuit voltage (OCV) to 0.40 V with hold times of 1 min/point. Cell internal resistance was measured at current densities above 100 mAcm^−2^ using the current interrupt technique with the load box and the Fuel Cell V.3.2 software (Scribner Associates Inc.). 

The total O_2_ mass transport resistance was derived from limiting current measurements at different total pressures and varied O_2_ concentrations [28,29,30]. High stoichiometric flow rates of the reactant gases were used to maintain uniform gas conditions in the flow channel. A 1.0 slpm flow of H_2_ was used at the anode, while 1.5 slpm mixtures of O_2_/N_2_ of varying oxidant concentrations were used at the cathode with O_2_ flow exceeding a stoichiometric ratio of 10 at all testing conditions. The cell temperature was maintained at 80 °C with inlet gases at 62% RH during measurements. Dry O_2_ mole fractions (1.0%, 1.5%, 2.0%, and 2.5%) in N_2_ were regulated from an ultra-high purity air tank equipped with a mass-flow controller (mks, Model M100B01322CS1BV). The anode and cathode total pressures (110 kPa_abs_, 150 kPa_abs_, 200 kPa_abs_, and 300 kPa_abs_) were varied to isolate pressure-dependent and pressure-independent O_2_ transport resistances. The cell voltage was scanned from 0.3 V to 0.06 V in 0.03 V steps, held 2 min at each voltage, and the limiting current was measured in each gas mixture and pressure. The analysis to calculate oxygen transport resistances followed the procedure outlined in the Results and Discussion section.

### 2.2. PEMFC Performance in Dead-Ended Anode (DEA) Mode

**Figure 1:**
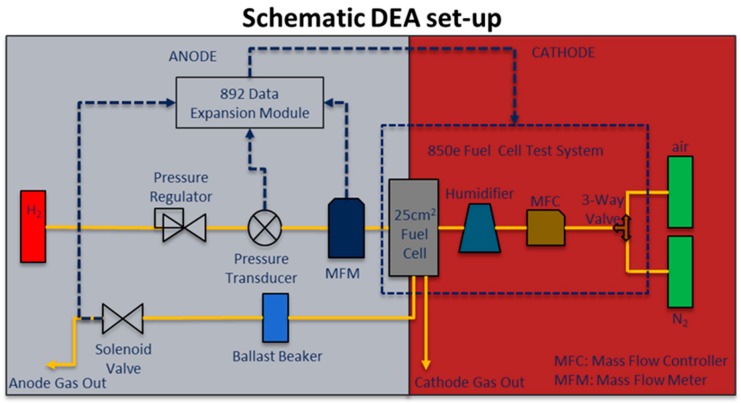
Experimental set-up schematic to test a single cell PEMFC in dead-ended anode (DEA) configuration.

Figure 1 presents a schematic of the experimental set-up used to test the PEMFCs in a dead-ended anode configuration.

In DEA mode, the anode compartment was fed with dry H_2_ from a gas cylinder. The hydrogen inlet pressure was controlled using a pressure regulator (Harris Mechanical Regulator) and monitored using a pressure transducer (Omega, part# PX359-015CG5V). The inlet H_2_ flow rate was measured using a mass-flow meter (Masterflex Differential Pressure Flowmeter, Item# EW-32908-69, Cole Palmer). Signals for the pressure transducer and mass flow rate were recorded using an 892e Data Expansion Module connected to the 850e Fuel Cell Test System (Scribner Associates, Inc.). A normally closed solenoid valve (P/N: 009-0631-900, Parker), controlled and activated by the 850e Fuel Cell Test System, was installed at the outlet of the anode to accomplish the DEA operation. On the cathode side, the 850e Fuel Cell Test System was used to control air flow rate (i.e., stoichiometric flow rate of 2) and air relative humidity (RH) supplied under ambient pressure. The humidifier was filled with 18 MΩ cm water from a Barnstead Nanopure System. The cell operating temperature was set to 65 °C. The inlet dew point for the air was set at 50.3 and 37 °C, corresponding to 50% and 25% RH_inlet_, respectively. Ultrapure air (UZ 300) and H_2_ (HY UHP 300) purchased from Air-liquide was supplied to the cathode and anode side, respectively.

To measure I-V polarization curves in DEA mode, we adapted a purging scheme from references [31,32,33] that entailed a 1 s duration purge every 60 s. The PEMFCs were operated at constant current (i.e., galvanostatic) mode with selected current densities of 200, 400, 600, 800, 1000, 1200, and 1400 mA cm^−2^. The anode compartment was fed with dry hydrogen. The anode H_2_ inlet pressure was set to 2 psi. After the load current was applied to the PEMFC, the solenoid valve was opened for 1 min to purge impurities inside the anode compartment and then closed for DEA mode. Humidified air was supplied to the cathode compartment under ambient pressure with a stoichiometric flow rate of 2 for all the current densities. The cell voltage was recorded and monitored by the Scribner 850e Fuel Cell Test System. All selected current densities were held for 3 min. During this time frame, the Scribner 850e Fuel Cell Test System sent a signal to open the solenoid every minute to purge the anode compartment for a set purging duration of 1 s and then the solenoid valve was closed again. Following this purge schedule, cell voltages did not fluctuate significantly during the DEA mode galvanostatic measurement, confirming that the PEMFCs had achieved steady-state equilibrium. 

The temporal evolution of the cell voltages was also investigated. The anode compartment was fed with dry H_2_. The anode H_2_ inlet pressure was set to 2 psi. Humidified air was supplied to the cathode compartment under ambient pressure with a stoichiometric flow rate of 2 for all the current densities. The PEMFCs were operated at two current densities, i.e., 800 mA cm^−2^ and 1200 mA cm^−2^. Initially, the PEMFC was operated at a selected current density in the flow-through mode. After the voltage was stabilized, the solenoid valve was closed. When the cell voltage dropped by 0.10 V, the solenoid valve was opened for 1 s. This purging cycle was repeated over a 50 min minimum period for each operating condition. 

## 3. Results and Discussion

### 3.1. PEMFC Performance in Regular Flow-Through Mode

**Figure 2:**
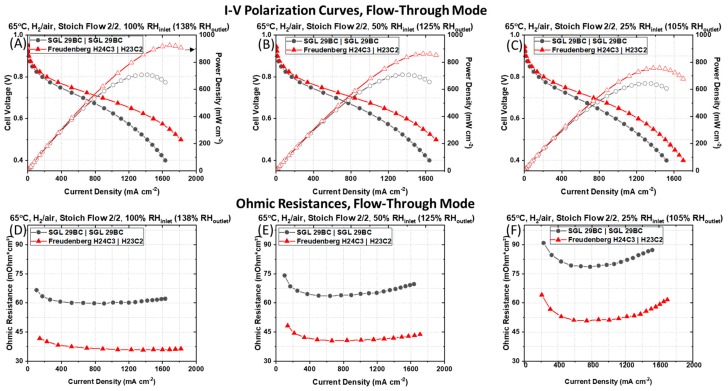
I–V polarization and power densities curves (**A**–**C**) with the associated Ohmic resistances (**D**–**F**) measured for a PEMFC containing a typical symmetric pairing of SGL 29BC GDM on the anode and cathode side and a PEMFC containing an asymmetrical pairing of Freudenberg GDM [i.e., H24C3 (anode) │ H23C2 (cathode)] recorded at a cell temperature of 65 °C, ambient pressure, in H_2_|air environment at stoichiometric flow rates of 2|2 at (**A**,**D**) 100%, (**B**,**E**) 50%_,_ and (**C**,**F**) 25% RH_inlet_, respectively, from left to right columns.

The PEMFC with the asymmetric GDM pairing has lower Ohmic resistances and significantly greater current and power densities at all operating conditions tested in flow-through mode, which flows H_2_ continuously at a fixed stoichiometric value relative to the air flow. Figure 2 shows the typical I–V polarization and power density curves (A, B, and C) with the associated Ohmic resistances (D, E, and F) measured for a PEMFC containing a symmetric pairing of SGL 29BC GDM on the anode and cathode side (black circle) to a PEMFC containing an asymmetric pairing of Freudenberg GDM on the anode (i.e., H24C3) and cathode (i.e., H23C2) side (red triangle) at a cell working temperature of 65 °C, fed with ambient pressure air, in H_2_|air at stoichiometric flow of 2/2 humidified at 100%, 50%, and 25% RH_inlet_, respectively. 

In the kinetic region of the polarization curve (i.e., operating cell voltage ≥ 0.80 V), there is a smaller effect of mass transport and current densities are very similar for both PEMFCs at all operating conditions. The differences in performance between the PEMFCs containing the symmetric SGL 29BC GDM pairing and the PEMFC containing the asymmetric Freudenberg GDM pairing are more pronounced at the lower cell operating voltage (i.e., operating cell voltage ≤ 0.60 V). Higher current densities are systematically measured for the PEMFC containing the asymmetric Freudenberg GDM pairing (i.e., H24C3 (anode) │ H23C2 (cathode)) at lower cell voltage requiring a higher consumption of O_2_, H^+^, and rejection of H_2_O, compared to the PEMFCs containing the symmetric SGL 29BC GDM pairing at all operating conditions. 

The current density measured at an operating cell voltage of 0.60 V increases from 1101 to 1394 mA cm^−2^ at 50% RH_inlet_ and from 976 to 1217 mA cm^−2^ at 25% RH_inlet_ when the symmetric GDM pairing of SGL 29BC is replaced by the asymmetric pairing of Freudenberg GDM. The benefit is even greater in more humidified gas flows, when the presence of liquid water is more certain, as we observe a 36% increase in the measured current density at 100% RH_inlet_ in the PEMFC with the asymmetric Freudenberg GDM pairing (i.e., from 1107 to 1491 mA cm^−2^). The peak power density increases by 31.5% at 100% RH_inlet,_ 22% at 50% RH_inlet_, and 18% at 25% RH_inlet,_ respectively, when the symmetric SGL 29BC GDM pairing is replaced by the asymmetric Freudenberg GDM pairing. 

As shown in Figure 2D–F, the Ohmic resistances measured at a current density of 1000 mA cm^−2^ for the PEMFC containing the symmetrical SGL 29BC GDM pairing were 60 mOhm cm^2^ at 100% RH_inlet,_ 64 mOhm cm^2^ at 50% RH_inlet_, and 80 mOhm cm^2^ at 25% RH_inlet_, respectively, compared to 36 mOhm cm^2^ at 100% RH_inlet,_ 42 mOhm cm^2^ at 50% RH_inlet_, and 51 mOhm cm^2^ at 25% RH_inlet_ for the PEMFC containing the asymmetric Freudenberg GDM pairing. The lower values of the cell Ohmic resistance for the PEMFC containing the asymmetric Freudenberg GDM pairing suggests that the cell is less sensitive to the adverse effects of dehydration in dry operating conditions. We describe these effects extensively in our prior work [26]. In short, Freudenberg GDM have a significantly lower compressibility, maintain a relatively large void volume at high levels of compressive stress, and have smoother MPL surfaces. These traits are expected to minimize cell contact resistances without sacrificing high gas transport.

**Figure 3:**
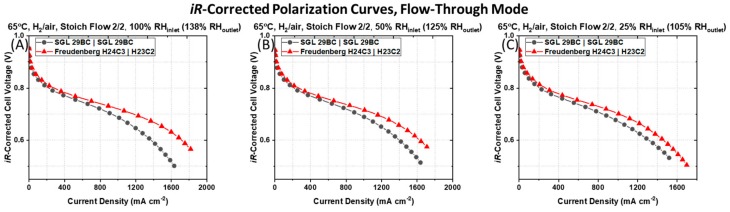
*iR*-corrected I–V polarization curves measured for a PEMFC containing a typical symmetric pairing of SGL 29BC GDM on the anode and cathode side and a PEMFC containing an asymmetric pairing of Freudenberg GDM (i.e., H24C3 (anode) │ H23C2 (cathode)) recorded at a cell temperature of 65 °C, ambient pressure, in H_2_|air environment at stoichiometric flow rates of 2|2 at (**A**) 100% RH_inlet_, (**B**) 50% RH_inlet_, and (**C**) 25% RH_inlet_, respectively.

The difference in the polarization behavior is not simply a function of the reduction of the cell Ohmic resistance for the PEMFC containing the asymmetric Freudenberg GDM pairng, as this GDM pairing still maintains a higher current density after *iR*-correcting the cell voltages, as shown in Figure 3. The *iR*-corrected polarization curves, which account for differences in cell Ohmic resistances, reveal that the PEMFC containing the symmetric SGL 29BC GDM pairing maintain lower current densities at all *iR*-corrected cell voltages compared to the PEMFC containing the asymmetric Freudenberg GDM pairing. This indicates that in addition to the differences in Ohmic resistance, there are additional sources of resistances in the PEMFCs with different GDM pairing.

To quantify the total oxygen transport resistance (R_total-O₂_), O_2_ limiting current measurements were performed. This experiment resolves where the liquid water is saturating to occlude gas transport. The R_total-O₂_ was calculated using Equation (1) [28] from the slope of the plot between the limiting current and dry O_2_ mole fraction at different total pressures as described in the experimental section.

(1) $$R_{Total-O_{2}}=\frac{4FC_{O_{2}}}{i_{lim}}=\frac{4F}{i_{lim}}\times \frac{P_{abs}-P_{H_{2}O}}{R\times T}\times x_{O_{2-dry}}$$

In Equation (1), C_O2_ is the gas channel O_2_ concentration, i_lim_ is the measured limiting current density (A cm^−2^_geometric_), F is the Faraday constant (96485 C mol^−1^), R is the universal gas constant (8.3145 J mol^−1^ K^−1^), T is the cell temperature (K), P_abs_ is the absolute gas pressure, and P_H₂O_ is the partial pressure of water. The R_total-O₂_ can be also be described by Equation (2) [30]:

(2) $$R_{Total-O_{2}}=R_{O_{2}}^{P-dep}+R_{O_{2}}^{P-ind}$$

where R_O₂_^P-dep^ is the pressure dependent O_2_ bulk diffusion resistance and R_O₂_^P-ind^ is the pressure independent oxygen transport resistance. R_O₂_^P-dep^ describes Fickian intermolecular gas diffusion through larger pores (>100 nm diameter), while R_O₂_^P-ind^ comprises Knudsen diffusion in smaller pores of the microporous layer and the catalyst layers (<100 nm diameter) as well as diffusion through the ionomer film covering the Pt particles.

**Figure 4:**
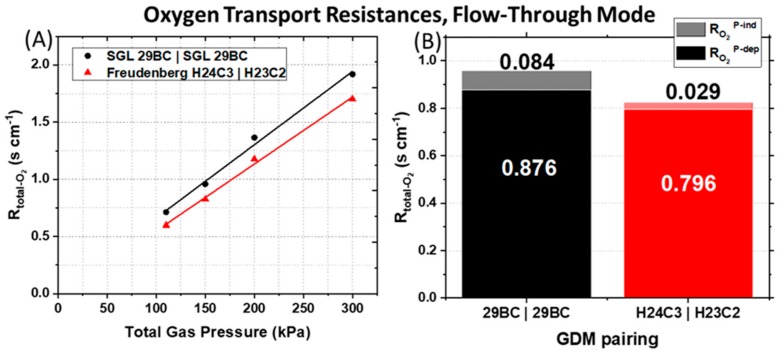
(**A**) Total O_2_ transport resistance (R_total-O₂_) calculated from Equation (1) as a function of total gas pressure. (**B**) Total O_2_ transport resistance calculated for the 150 kPa_abs_ back-pressure data set (R_total-O₂_ = sum of the solid and shaded bars) which can be separated into pressure dependent (R_O₂_^P-dep^) and pressure independent (R_O₂_^P-ind^) terms as described by Equation (2).

The asymmetric Freudenberg GDM pairing reduces both the pressure-dependent and pressure-independent O_2_ transport resistances, suggesting a decrease in liquid water saturation in the cathode. The calculated *R_total-O₂_* are plotted in Figure 4A as a function of the total gas pressure. We observe that both PEMFCs tested have a decrease in R_total-O₂_ with a decrease of the total gas pressure. Figure 4A shows the effect of the GDM pairing on the R_total-O₂_, which is the sum of the *R_O₂_^P-dep^* (solid bars) and *R_O₂_^P-ind^* (shaded bars) calculated for the 150 kPa_abs_ back-pressure data set. The symmetric anode and cathode GDM pairing (SGL 29BC) yields a R_O₂_^P-ind^ that is ~3× larger and R_O₂_^P-dep^ that is ~1.1× larger than the asymmetric Freudenberg GDM pairing.

This indicates that in the PEMFC with the symmetric SGL 29BC GDM pairing, oxygen diffusion is hindered by poor gas transport in the two pore size regimes: the fine pores in the cathode CL also including the ionomer or internal water (R_O₂_^P-ind^), as well as in larger pores such as those in the MPL, GDL, and the gas channel (R_O₂_^P-dep^). The exact form of water is uncertain, but possibilities include continuous or discontinuous film and droplets. The higher R_O₂_^P-ind^ can arise from either more water saturation in the cathode catalyst layer or from a very poorly hydrated cathode CL ionomer that impedes oxygen diffusion through the ionomer film to the active sites [28,34,35]. The accompanying high R_O₂_^P-dep^ suggests a greater oxygen diffusion resistance through the larger GDL or MPL pores or the gas channels, which is likely the result of the higher tortuosity in the GDL of SGL 29BC, which Zenyuk et al. [36] have reported to be several times greater than the tortuosity of the GDL region of the Freudenberg H23C2 used at the cathode in the asymmetric GDM pairing. 

Compared to the symmetric SGL 29BC GDM pairing, the asymmetric Freudenberg GDM pairing overall displays superior power, presumably due to better management of the water, reactants and products. The latter GDM configuration can effectively remove significant amounts of liquid water for high humidity and high power operation as well as maintain membrane hydration during dry operation. Considering that the PEMFC with asymmetric Freudenberg GDM pairing has lower Ohmic resistances (Figure 2D–F) and lower oxygen transport resistances (Figure 4), we conclude that this asymmetric Freudenberg GDM pairing promotes more effective water management in flow-through mode.

### 3.2. PEMFC Performance in Dead-Ended Anode (DEA) Mode

The PEMFC with the asymmetric GDM pairing promotes more effective water management in flow-through mode (Section 2.1), and we postulate that this GDM selection will also enhance PEMFC performance in DEA mode and resolve voltage decay due to excessive accumulation of back-diffused liquid water from the cathode and dilution of the anode fuel concentration via N_2_ crossover. During the DEA operation of a PEMFC, when air is supplied as the oxidant, water produced at the cathode back-diffuses across the membrane to the dry anode and accumulates in the anode flow channel [7,8,9,10,11,12]. This accumulation of water at the anode blocks the gas transport pathway. Additionally, nitrogen may be transported through the membrane from cathode to anode due to pressure and concentration gradients [7,12,13,14,15], resulting in local fuel (i.e., H_2_) starvation and performance loss. Cell voltage decay in this study will have a stronger sensitivity to the former failure mode, anode water accumulation from insufficient water management, because these PEMFCs only differ in GDM selection. We assume that the N_2_ crossover rate will not change for the two PEMFCs tested because they are comprised of identical catalyst coated membranes (CCMs).

**Figure 5:**
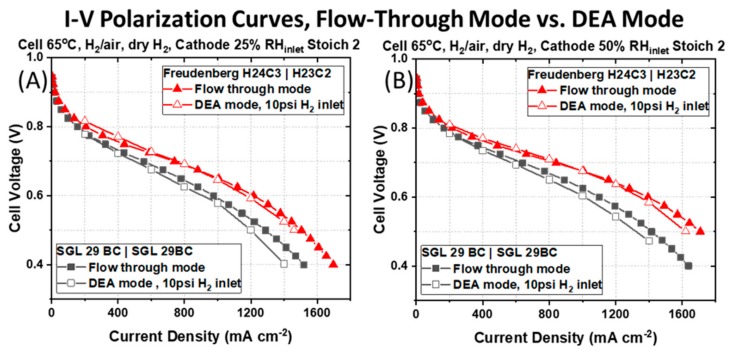
Galvanostatic I–V polarization curves measured for a PEMFC containing a typical symmetric pairing of SGL 29BC GDM on the anode and cathode side and a PEMFC containing an asymmetric pairing of Freudenberg GDM. Polarization curves are measured in DEA mode (open symbols) and flow-through mode (closed symbols) at (**A**) 25% RH_inlet_ cathode and (**B**) 50% RH_inlet_ cathode. In all cases, the cell temperature is 65 °C, in H_2_|air, with air supplied to the cathode at stoichiometric ratio of 2 at atmospheric pressure. During DEA mode, dry H_2_ is supplied to the anode at 2 psi. In flow-through mode, the inlet RH of the H_2_ at the anode matches the cathode RH and H_2_ flows at a stoichiometric ratio of 2.

The advantage of using asymmetric GDM observed in flow-through mode is maintained when the PEMFCs are operated in DEA mode. Polarization curves for the two PEMFCs with different GDM pairings are shown with open symbols in Figure 5.

Owing to the pressure and concentration gradients between anode and cathode during DEA mode, we anticipate higher amounts of water back-diffusion across the membrane, and greater liquid water saturation at the anode. That the asymmetric GDM pairing maintains higher cell voltages and power compared to the PEMFC with symmetric GDM during DEA mode supports that an asymmetric GDM pairing imparts superior water management. At 800 mA cm^−2^ and 25% RH_inlet_ at the cathode [Figure. 5(A)], the PEMFC with asymmetric GDM (Freudenberg H24C3 | H23C2, red curve) pairing produces 0.67 V compared to 0.61 V for the PEMFC with symmetric GDM (SGL 29BC). Similarly, a considerable increase in voltage (and power) is measured at 1200 mA cm^−2^, 0.57 to 0.48 V, when an asymmetric GDM pairing is used in place of a symmetric GDM pairing commonly reported to be used in the open literature. The same trends are observed with a more humidified cathode gas stream in Figure 5B. There are marginal decreases in cell voltages during operation in DEA mode compared to flow-through mode (~42 mV at 1200 mA cm^−2^) for both PEMFCs under study. These are attributed to the use of dry hydrogen on the anode side during testing in DEA mode, while in flow-through mode, the anode RH_inlet_ matches that at the cathode.

**Figure 6:**
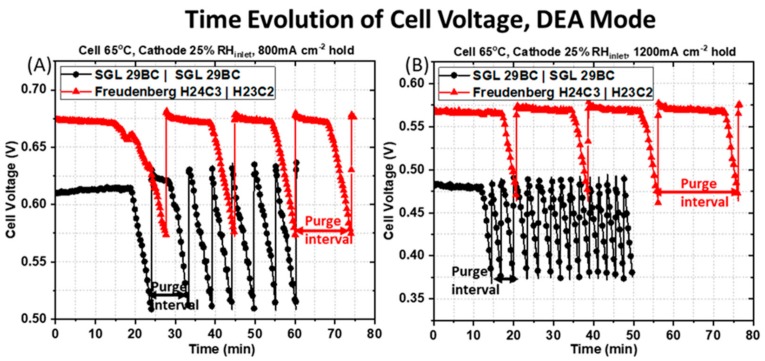
Time evolution of the cell voltage obtained at applied current densities of (**A**) 800 mA cm^−2^ and (**B**) 1200 mA cm^−2^ for an H_2_ inlet pressure of 2 psi and 25% RH_inlet_ cathode. The purge interval is defined as the time between each valve opening event; examples of a purge interval are depicted by the black and red arrows at the bottom of the figures.

The time evolution of the cell voltage is significantly different for the PEMFC containing the symmetric SGL 29BC GDM pairing and the PEMFC containing the asymmetric Freudenberg GDM pairing. Figure 6 shows the time evolution of the cell voltage obtained in DEA mode at two applied current densities of (A) 800 mA cm^−2^ and (B) 1200 mA cm^−2^ for a H_2_ inlet pressure of 2 psi and with 25% RH_inlet_ air flowing at the cathode. The purge interval is defined as the time between each valve opening event. Examples of these are illustrated at the bottom of Figure 6A,B for each pairing of GDM by either the black or red arrows. The criterion for triggering a valve-opening event was a voltage decay of 0.10 V during constant current operation. All of these observations support that water accumulates in the anode faster for a PEMFC with a symmetric GDM pairing. First, the time between purge events is much greater for the asymmetric GDM pairing. It requires more time at a given current density for the anode to saturate with water and cell voltage decay to be observed. The asymmetric GDM spends a longer time at steady-state prior to voltage decay, and its periods of voltage decay are more gradual. Further, the applied current density has a major impact on the purge interval for each of the PEMFCs under study. A reduction of the purge interval with increasing current density, from Figure 6A to Figure 6B, highlights the inferior water management of the symmetric GDM pairing (black circles) compared to the asymmetric GDM pairing (red triangles). A longer purge duration is desirable in a fuel cell system because it increases H_2_ utilization and reduces valve wear.

As shown in Figure 6A for the PEMFC containing the symmetric SGL 29BC GDM pairing (black circles) at the applied current density of 800 mA cm^−2^ the cell output voltage is maintained at a constant value of ~0.61 V initially from 0 min to 20 min 37 s. This behavior represents an absence of liquid water in the GDL and the channels at the beginning of the experiment after dry H_2_ was purged through the anode compartment and prior to water production at the cathode. The cell operating voltage drops rapidly after 20 min 37 s, likely due to liquid water saturation in the anode GDM or flow fields from back-diffused water. After 3 min 21 s of steady voltage decline, the first purge event happens and the solenoid is opened to remove water/impurities accumulated in the anode. After the 1 s gas purging duration, the cell operating voltage rises and recovers rapidly to its original level, implying that the 1 s purging duration is sufficient to discharge the accumulated water at this applied current density of at 800 mA cm^−2^. The cell output voltage is stable again for ~7 min, and then steadily declines for about 3 min 21 s, when the next purge event happens. As the experiment progresses, the gap between each purge intervals becomes shorter. There is a mean value of 2 min 24 s ± 21 s for the constant voltage hold period (steady-state, prior to the rapid voltage decay) and a mean value of 2 min 58 s ± 18 s for the regions of rapid voltage decline. This sums to a mean purge interval of 5 min 22 s ± 22 s for the total of five purge events for the last 24 min of the experiment.

For the same operating condition, the response obtained for the PEMFC containing the asymmetric Freudenberg GDM pairing is drastically different, as shown in Figure 6A. The average purge time for the PEMFC with asymmetric GDM is extended by ~10 min compared to the PEMFC with symmetric GDM that are more prone to anode water saturation. The cell output voltage is maintained at a constant value of 0.67 V initially from 0 min to 17 min 46 s, again probably representing an absence of liquid water in the GDL and the channels during this period after dry hydrogen was purged through the anode compartment prior to current generation. The cell operating voltage begins to drop smoothly after 17 min 46 s, likely due to the initial saturation by back-diffused water. The cell voltage decays relatively slowly, ~10 min of steady cell output decline, before the first purge event happens. As the experiment progresses, the gap between each purge intervals remains constant, with mean value of 10 min 45 s ± 1 min 27 s for the constant voltage hold period and a mean value of 4 min 42 s ± 10 s for the steady voltage decline period. This corresponds to mean purge interval of 15 min 27 s ± 1 min 33 s for a total of three purge events for the last 47 min of the experiment.

As the applied current density increases from 800 to 1200 mA cm^−2^, the water generated in the cathode becomes larger, and there is an expected increase in the water back-diffused from the cathode to the anode due to the difference in water vapor concentration. Therefore, the accumulated water in the anode compartment becomes larger as the current density increases. This is reflected in the cell voltage response shown in Figure 6B for the PEMFC containing the symmetric SGL 29 BC GDM pairing at 1200 mA cm^−2^ and 2 psi H_2_ inlet pressure. The cell voltage is maintained at a constant value of ~ 0.48 V initially from 0 min to 13 min 25s, again probably representing an absence of liquid water in the GDL and the channels during this period, where dry hydrogen is purged through the anode compartment. The cell operating voltage begins to drop smoothly after 13 min 25s, likely due to the initial saturation of back diffusive water vapor. After ~1 min of steady cell output decline, the first purge event happens, the solenoid is opened to remove water/impurities accumulated in the anode compartment. After the 1 s gas purging duration, the cell operating voltage rose and recovered rapidly to its original level, implying that the 1 s purging duration is sufficient to discharge the accumulated water at this applied current density of at 1200 mA cm^−2^. However, as the experiment progresses, the gap between each purge interval is very stable and brief, with a mean value of 1 min 30 s ± 26 s for the constant voltage hold period and a mean value of 1 min ± 3 s for the steady voltage decline period. This corresponds to a mean purge interval of 2 min 33 s± 28 s for a total of 13 purge events for the last 33 min of the experiment. This is a significant increase in purge frequency compared to the lower current density operation.

The PEMFC containing the asymmetric Freudenberg GDM pairing has a different behavior, as shown in Figure 6B. Unlike with the symmetric GDM pairing, which saw a reduction in purge interval at higher current density, the average purge interval marginally increases at higher current density when asymmetric GDM are used. The cell voltage is maintained at a constant value of 0.57 V initially from 0 min to 17 min 34 s, again probably representing an absence of liquid water in the GDM and the channels during this period, where dry hydrogen is purged through the anode compartment. The cell operating voltage begins to drop smoothly after 17 min 34 s, likely due to the initial saturation of back-diffused water vapor. After ~3 min 10 s of steady cell output decline, the first purge event happens, the solenoid is opened to remove water/impurities accumulated in the anode compartment. As the experiment progresses, the gap between each purge interval remains stable, with a mean value of 15 min 14 s ± 1 min 40 s for the constant voltage hold period and a mean value of 3 min 16 s ± 20 s for the steady voltage decline period. This corresponds to a mean purge interval of 18 min 29 s ± 1 min 21 s for a total of three purge events for the last 55 min of the experiment.

**Figure 7:**
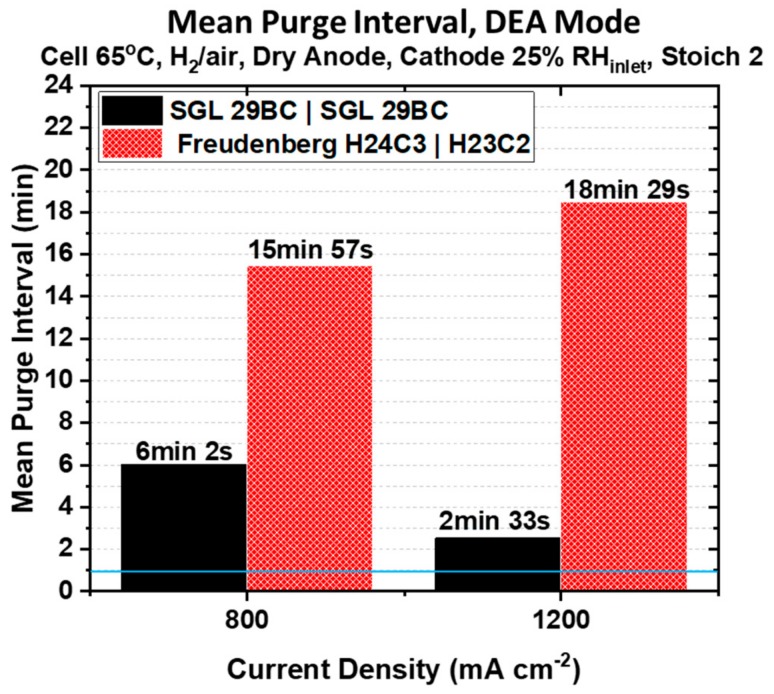
Variation of the mean purge interval as a function of the applied current density. The horizontal blue line at 1 min marks the purge duration used to collect the polarization curves reported in Figure 5.

Figure 7 compares the variation of the mean purge interval observed for the data in Figure 6 as a function of the fixed current density for both PEMFCs under study when testing in steady state DEA mode. There is a longer time before the first purge is required due to the initial absence of liquid water in the GDM and the channels prior to the start of the DEA mode operation, as dry hydrogen is purged through the anode compartment. It should be noted that the first purge interval is not included in this calculation because the PEMFC has not yet reached an equilibrium state in hydration. For reference, the blue line shows the purge interval used during polarization characterization in Figure 5.

Superior water management imparted by an asymmetric GDM pairing will conserve H_2_ fuel by vastly reducing anode purging frequency, another advantage of using the Freudenberg asymmetric GDM pairing for a PEMFC in DEA mode. At the applied current density of 800 mA cm^−2^, the mean purge interval is 2.7× longer for the PEMFC containing the asymmetric Freudenberg GDM pairing. As shown in Figure 6A, only four purging events are necessary over an 80 min period compared to seven over a 60 min period for the PEMFC containing the symmetric SGL 29BC pairing. As the applied current density increases from 800 to 1200 mA cm^−2^, the water generated in the cathode increases and we expect a greater back-diffusion driving force from the cathode to the anode due to the greater difference in water vapor concentration between the two electrodes. Therefore, we expect greater water accumulation in the anode compartment as the current density increases. It can be seen from Figure 7, that the PEMFC containing the symmetric SGL 29BC pairing is the most affected by the increase in the current density. The mean purge interval is more than 50% shorter, and a total of fourteen purge events are necessary over a 50 min period compared to only four in an 80 min period for the PEMFC containing the Freudenberg asymmetric GDM pairing, as shown in Figure 6B. The PEMFC constructed with the asymmetric Freudenberg GDM pairing requires even less frequent anode purging at higher current densities. We attribute this to an expected difference in thermal conductivity for the two cathode GDM used in these PEMFCs. Fibrous GDL substrates from Freudenberg have been shown to have a lower thermal conductivity than those GDL substrates developed by SGL [37,38]. With a lower thermal conductivity in the cathode GDL substrate, the cathode catalyst layer temperature would increase, facilitating product water evaporation for rejection from the cathode.

## 4. Conclusions

PEMFCs with an asymmetric Freudenberg GDM pairing that feature different GDM at the anode and the cathode have superior I–V polarization curves in flow-through mode as compared to PEMFCs with a typical symmetric SGL 29BC GDM pairing (i.e., the same GDM is used on the anode and cathode side of the MEA). Compared to the symmetric SGL 29BC GDM pairing, the asymmetric Freudenberg GDM pairing overall displays superior water management. The PEMFC with the asymmetric GDM pairing has lower Ohmic resistances and significantly greater current and power densities at all operating conditions tested in flow-through mode. Additionally, the asymmetric GDM pairing reduces oxygen transport resistances. This result coupled with the significantly lower ohmic resistance suggests that the asymmetric GDM pairing hydrates the membrane and catalyst layers without excess liquid water saturation at the cathode. 

The advantage of using asymmetric GDM observed in flow-through mode is maintained when the PEMFCs are operated in DEA mode, supporting that an asymmetric GDM pairing imparts superior water management, even when the driving force for water back-diffusion to the anode is enhanced. This is manifested during DEA mode in several ways. First, the cell voltage is consistently greater at all current densities at both 25% and 50% cathode inlet RH. Second, the asymmetric GDM pairing significantly increases the mean time required between anode purge events. It requires significantly more time at a given current density for the anode to saturate with water and cell voltage decay to be observed. 

This promises H_2_ fuel conservation for a PEMFC stack comprised of asymmetric GDM pairings. Additionally, the asymmetric GDM spends longer time at steady-state prior to voltage decays, and its periods of voltage decay are more gradual. The applied current density has a unique impact on the purge interval for each of the PEMFCs under study. At the applied current density of 800 mA cm^−2^, the mean purge interval is 2.7× longer for the PEMFC containing the asymmetric Freudenberg GDM pairing. The PEMFC containing the symmetric SGL 29BC pairing is the most affected by the increase in the current density. After increasing the current density from 800 to 1200 mA cm^−2^, the mean purge interval is more than 50% shorter, and a total of fourteen purge events are necessary over a 50 min period compared to only four in an 80 min period for the PEMFC containing the Freudenberg asymmetric GDM pairing at the higher current density. The PEMFC constructed with the asymmetric Freudenberg GDM pairing requires even less frequent anode purging at higher current densities. The PEMFC with symmetric GDM pairing must be purged 7.4× more often at this current density.
